# Machine learning constructs a diagnostic prediction model for calculous pyonephrosis

**DOI:** 10.1007/s00240-024-01587-y

**Published:** 2024-06-19

**Authors:** Bin Yang, Jiao Zhong, Yalin Yang, Jin Xu, Hua Liu, Jianhe Liu

**Affiliations:** 1 Department of Urology, The Second Affiliated Hospital of Kunming Medical University, NO. 374 Dianmian Avenue, Wuhua District, Kunming, 650101 China; 2 Department of Urology, The Second People’s Hospital of Yibin City, No. 96, North Street, Yibin, 644000 China

**Keywords:** Machine learning, Pyonephrosis, Upper urinary tract calculi, Prediction model, Diagnosis

## Abstract

In order to provide decision-making support for the auxiliary diagnosis and individualized treatment of calculous pyonephrosis, the study aims to analyze the clinical features of the condition, investigate its risk factors, and develop a prediction model of the condition using machine learning techniques. A retrospective analysis was conducted on the clinical data of 268 patients with calculous renal pelvic effusion who underwent ultrasonography-guided percutaneous renal puncture and drainage in our hospital during January 2018 to December 2022. The patients were included into two groups, one for pyonephrosis and the other for hydronephrosis. At a random ratio of 7:3, the research cohort was split into training and testing data sets. Single factor analysis was utilized to examine the 43 characteristics of the hydronephrosis group and the pyonephrosis group using the T test, Spearman rank correlation test and chi-square test. Disparities in the characteristic distributions between the two groups in the training and test sets were noted. The features were filtered using the minimal absolute value shrinkage and selection operator on the training set of data. Auxiliary diagnostic prediction models were established using the following five machine learning (ML) algorithms: random forest (RF), xtreme gradient boosting (XGBoost), support vector machines (SVM), gradient boosting decision trees (GBDT) and logistic regression (LR). The area under the curve (AUC) was used to compare the performance, and the best model was chosen. The decision curve was used to evaluate the clinical practicability of the models. The models with the greatest AUC in the training dataset were RF (1.000), followed by XGBoost (0.999), GBDT (0.977), and SVM (0.971). The lowest AUC was obtained by LR (0.938). With the greatest AUC in the test dataset going to GBDT (0.967), followed by LR (0.957), XGBoost (0.950), SVM (0.939) and RF (0.924). LR, GBDT and RF models had the highest accuracy were 0.873, followed by SVM, and the lowest was XGBoost. Out of the five models, the LR model had the best sensitivity and specificity is 0.923 and 0.887. The GBDT model had the highest AUC among the five models of calculous pyonephrosis developed using the ML, followed by the LR model. The LR model was considered be the best prediction model when combined with clinical operability. As it comes to diagnosing pyonephrosis, the LR model was more credible and had better prediction accuracy than common analysis approaches. Its nomogram can be used as an additional non-invasive diagnostic technique.

## Introduction

Urolithiasis has been reported to be on the rise in both adults and children in recent decades [1, 2]. About 75% of cases of hydronephrosis are caused by upper urinary tract calculi, which are followed by ureteral strictures and tumors [3]. Pyonephrosis can result from urinary tract infections that are easily caused by obstructive hydronephrosis [4]. The presence of pus in a blocked renal collecting system is known as pyonephrosis [5]. Acute renal empyema patients may have a variety of clinical symptoms, such as fever, or abnormal laboratory results, such as elevated white blood cell counts, procalcitonin, noticeably elevated urine white blood cell counts, or urine cultures that are positive when brought to the doctor’s attention promptly. Some individuals with persistent infections, however, frequently show no symptoms as all and have significantly abnormal laboratory test results. This could hinder these patients from being identified in a timely manner and worsen kidney damage. In a brief period of time, 85% of patients may develop sepsis-related serious complications including urosepsis [6]. Septic shock has up to a 50% fatality rate, which is extremely detrimental to both individuals and society [7]. As a consequence, the key to arresting the progression of renal empyema is the early identification [8, 9].

Though they have constraints, the findings of imaging from computed tomography (CT), magnetic resonance imaging (MRI), and ultrasound can help discriminate between pyonephrosis and hydronephrosis. Along with contributing to rapidly confirming or determining out the presence of hydronephrosis in the renal pelvis, ultrasound, a traditional preliminary imaging methodology, can also reveal echogenic debris in the dilated collection system, which is thought to be a strong indicator of pyonephrosis in the renal pelvis [10], though it is not a specific sign [11, 12].

Numerous prior studies have investigated HU value of effusion in differentiating between the types of renal pelvic effusion. For instance, as significant independent predictors of infection, Gnannt utilized diabetes, C-reactive protein (CRP), HU value of effusion, and intracavitary gas [13]. Pyonephrosis is diagnosed by expanded collection system on enhanced CT scan, thickness of the renal pelvis wall (> 2 mm), and inflammatory alterations in the perirenal fat [14, 15]. Under magnetic resonance diffusion weighted imaging (DWI), the apparent diffusion coefficient (ADC) value of pyonephrosis was significantly less than that of non-infectious hydronephrosis [16]. These indicators, however, are frequently not diagnostic for any patient. It will also be challenging to diagnose the patient if they lack apparent symptoms [17].

Two criteria must be come across for the diagnosis of calculous pyonephrosis. Initially, upper urinary tract stones, meaning that all patients experienced different degrees of renal, ureteral, or renal ureteral stones confirmed by CT scan. Secondly, pus or purulent aggregates with varying viscosities seen with the naked eye during ultrasound-guided percutaneous renal (effusion) puncture and drainage [5]. Puncture drainage, however, is an intrusive procedure that might result in implications such bleeding or worsened infection.

Non-invasive diagnostic prediction models for clinical diagnosis and therapy have been constantly developed with the advent of machine learning (ML) in the medical arena. ML is a fast-growing area within computer science. It mimics the identification, analysis, problem-solving, and other cognitive processes of humans. The sophisticated ML technique enables it to handle intricate situations that conventional models are unable to manage. Wang et al [18] designed a prediction model by screening characteristics from 22 clinical indicators and retrospectively analyzing the clinical data of 322 patients with renal pelvic effusion complicated by calculi. With an area under the curve (AUC) of 0.981, they revealed that the XGBoost model performed the best in terms of predictions on the training set. With an AUC of 0.977, the SVM model performs the best on the test set, the diagnostic accuracy is much higher than with the conventional statistical analysis method.

With the goal to provide decision-making support for the auxiliary diagnosis and customized treatment of calculous pyonephrosis, the aim of this work is to analyze the clinical features of the condition while developing a diagnostic prediction model using ML techniques.

## Methods

### Patient selection and study parameters

The ethical review committee of our institution has authorized this single-center retrospective study. Retrospective analysis was done on the medical records of 268 patients with renal pelvic effusion and upper urinary calculi who received ultrasound-guided percutaneous renal (fluid) puncture while in the hospital during January 2018 and December 2022.

The following are the inclusion criteria: patient must be not less than eighteen years old; patients must have upper urinary tract calculi (ureteral or renal calculi) that lead to renal pelvis effusion; our hospital provides ultrasound-guided percutaneous puncture and renal effusion drainage; all clinical data, including laboratory results, signs and symptoms and a non-enhanced CT scan before puncture.

These criteria were applied to exclude patients: non-enhanced CT examination images from other hospitals; ureteral stent implantation or puncture drainage prior to fistula substitute puncture; bilateral puncture drainage at which the urine on one side was clear and the other purulent; contrast agent interference or effusion that is not apparent and cannot be measured by HU value of effusion; combined with extra illnesses: lower urinary tract blockage, such as benign prostatic hyperplasia, bladder stones and urethral stricture; congenital urinary system anomalies, including as ectopic kidney, duplex kidney, solitary kidney and horseshoe kidney.

Gender, age, height, weight, body mass index (BMI), history of diabetes, hypertension, fever, smoking, drinking, ipsilateral stone surgery and renal colic are among the general clinical characteristics that are collected for clinical data. Features of the laboratory: Blood cell analysis and infection: hemoglobin, interleukin-6 (IL-6), CRP, procalcitonin (PCT), white blood cells (WBC), neutrophils, lymphocytes, neutrophil/lymphocyte ratio (N/L), red blood cells (RBC). Urine: urine WBC, urine RBC, urine culture, PH, urine bacteria, nitrite and leukocyte esterase. Blood biochemistry: albumin, globulin, globulin/albumin ratio (G/A), uric acid, creatinine, urea, blood glucose, triglyceride and cholesterol.

### Data processing

Once the clinical data acquiring, information were examined, rectified, and aberrant values were changed. For instance, the urine WBC count NEG was changed to fall between normal ranges. The K-nearest neighbors (KNN) technique is utilized to fill in the missing data. To enable it to more accurately represent the underlying structure of the data and the relationships between samples, the KNN algorithm takes into account the similarity between samples and fills in the missing values using information from the samples that are closest to each other. Only the general distribution features of the variables are taken into consideration, as opposed to the mode and mean filling; the correlation between samples is not taken into account either [19].

### Dataset splitting and feature screening

The dataset had been arbitrarily divided into two different subsets for this study: 70% for the training dataset and 30% for the model test dataset. A fixed number of random seeds was also given. Forty-three features in the training set were screened employing the least absolute shrinkage and selection operator (Lasso).

### Construction of five ML prediction models

The model was constructed utilizing R software, and the training dataset was used. Logistic regression (LR), eXtreme gradient boosting (XGBoost), support vector machines (SVM), random forest (RF) and gradient boosting decision trees (GBDT) were the five ML methods that were utilized for constructing prediction models.

Originally proposed by Robert Tibshirani [20], the Lasso regression utilized for feature screening. With Lasso regression, the most essential features can be identified. For the reason that to achieve sparsity, the optimization objective function is modified by introducing a penalty term causing the weight of many characteristics in the coefficient vector to fall to 0. The features with the highest predictive power for the target characteristics may be filtered away by choosing the features that correspond to the non-zero coefficients, which will simplify the model and improve its capacity for generalization [21].

LR is a parametric statistical model that mainly utilizes predictive data to estimate event probability. A common strategy for handling problems with classification, typically binary classification issues, in ML and statistics. It’s actually a classification technique, hence the name “regression.” The Sigmoid function is a way to mathematically map the outcomes of linear regression to probability values. Each prediction feature’s likelihood of developing is then calculated by multiplying it by the corresponding numerical parameters, and the LR function is deployed to distill all of the results [22].

XGBoost is an ensemble learning algorithm that is precise, versatile, and efficient. It makes use of decision trees as its core learner. The approach contains a built-in regularization mechanism to lessen overfitting, an outstanding built-in split search method to enhance the tree, and is around 10 times quicker than the conventional gradient lifting methodology. When it comes to tabular data, this lifting-based method outperforms other ML techniques. It primarily serves as a tool for tabular data and is considered as the most advanced tree-based approach [23].

SVM is a supervised ML approach for regression analysis and classification that is based on kernel tricks. It is frequently employed to handle an array of challenging data analysis tasks. Its main objective is to locate a decision hyperplane, or, more precisely, a geometric plane (represented as a straight line in two-dimensional space, a plane in three-dimensional space, and a hyperplane in high-dimensional space) in the feature space of multiple dimensions that can divide multiple kinds of data points in the most efficient way for performing classification tasks. The elements of SVM involve the Gaussian radial basis function (RBF) kernel, the polynomial kernel, the linear kernel, and the sigmoid kernel [24, 25].

Regression and classification are two typical applications for the ensemble learning technique known as RF. by generating many decision trees and exporting each tree’s pattern work. For determining the classification tree nodes, random forest simultaneously chooses a random subset of features from k self-feature choices. The classification tree that is created in this manner may vary from time to time. In general, dozens to hundreds of thousands of classification trees are produced at random via random forests, and the tree with the highest degree of recurrence is selected as the winner [26].

An ensemble learning algorithm that is a part of the decision tree ensemble approach is called GBDT. It improves the performance of the entire model by training a series of decision trees one after the other and iteratively correcting the prediction error of each tree. The benefit of GBDT is that it functions well with a wide range of datasets and may be used to solve classification and regression issues. The gradient of the gradient descent theory is the source of the GBDT’s “gradient.” To lower the loss function’s value, a new model is created in each iteration in the loss function’s negative gradient direction. To prevent overfitting and regulate the weight of the new model in each iteration, GBDT incorporates regularization and learning rate parameters [27].

### Validation of ML models

On various training datasets, the performance of any algorithm for prediction will vary. The model’s capacity to discriminate on the test dataset validates each model’s outcome. We choose the most suitable model based on clinical practicability, subsequently proceed with creating the calibration curve, execute decision curve analysis (DCA), and assess the model’s calibration, risk, and utility.

The model’s capacity to discern between various outcomes, as measured by the model fitting effect index. By calculating the AUC of the receiver operator characteristic curve (ROC) and discriminant indices such accuracy, sensitivity and specificity, the discriminant ability of each ML prediction model was assessed. We compared the ROC with the DeLong [28]. A measure of the model’s quality of fit that compares the projected probability of the result to the actual probability of the event’s occurrence. The Hosmer-Lemeshow goodness of fit test findings are plotted as a calibration curve, which is a scatter plot of the actual occurrence probability vs. the predicted occurrence probability. The technique to generate DCA curves to weigh the advantages and risks for patients while evaluating the viability of clinical decision-making.

R software was used for statistical analyses 43 features of 268 patients were tested for normality using the Kolmogorov-Smirnov, and the independent sample T test was performed to ensure that the data met expectations for a normal distribution. The Mann-Whitney rank sum test is performed if the data are not regularly distributed. The chi-square is used to determine categorization characteristics. It was determined to be statistically significant while *p* < 0.05.

The following R packages were used: ‘corrplot’, ‘glmnet’, ‘caret’, ‘CBCgrps’, ‘nortest’, ‘tidyverse’, ‘ggpubr’, ‘rms’, ‘PROC’, ‘ggplot2’, ‘compareGroups’, ‘DiagrammeR’, ‘e1071’, ‘rrtable’, ‘autoReg’, ‘Table 1’, ‘Hmisc’, ‘reportROC’, ‘randomForest’, ‘rmda’, ‘gbm3’, ‘Matrix’, and ‘xgboost ‘.

## Results

### General clinical characteristics

268 patients with renal pelvis effusion and upper urinary calculi who underwent ultrasound-guided percutaneous renal puncture while hospitalized were included in this study, which adhered strictly to the inclusion and exclusion criteria. The patients were found in the urinary calculi database between January 2018 and December 2022. The general clinical features of every participant in the study are listed in Table 1. The hydronephrosis group (*n* = 179) and the pyonephrosis group (*n* = 89) included 268 individuals with calculous hydronephrosis. There were 63 patients with pyonephrosis and 126 individuals with hydronephrosis in the training set (*n* = 189). There were 26 patients with pyonephrosis and 53 individuals with hydronephrosis in the testing set (*n* = 79).

26 men, or 41% of the training set, were in the pyonephrosis group. 37 women, or 59%, were in the same group. There were 55.46 years on average. The data revealed that 17% with diabetes, 27% with hypertension, 44% with history of ipsilateral stone surgery, 13% with renal colic, and 37% had fever. In the hydronephrosis group, 42 women made up 33% of the total, with 84 males making up 67% of the group. 52.91 was the average age. 5% with diabetes, 41% with hypertension, 37% with a history of ipsilateral stone surgery, 77% had renal colic and 18% with fever.

In the training set, two groups differed significantly in terms of gender (*p* = 0.001), diabetes (*p* = 0.009), fever (*p* < 0.001) and renal colic (*p* < 0.001), while gender (*p* = 0.004), fever (*p* = 0.007) and renal colic (*p* < 0.001) in the testing set.

**Table 1:** Comparison results of general clinical characteristics on two groups

Characteristic	Training set (*n* = 189)				Testing set (*n* = 79)		
	Hydronephrosis (*n* = 126)	Pyonephrosis (*n* = 63)	*p*		Hydronephrosis (*n* = 53)	Pyonephrosis (*n* = 26)	*p*
Age (year)	52.91 ± 12.8	55.46 ± 11.54	0.17		53.09 ± 12.03	57.23 ± 13.58	0.194
Weight (Kg) (IQR)	62 (54.25, 71.75)	60 (51.5, 70)	0.169		63 (53, 75)	55 (50.25, 61)	0.072
Height (mm) (IQR)	165 (156, 170)	160 (155, 167.5)	0.059		160 (157, 168)	158 (155, 165)	0.122
BMI (kg/m^2^) (IQR)	22.94 (21.03, 26)	23.44 (20.82, 5.53)	0.661		24.24 (20.57, 27.28)	22.31 (20.63, 24.97)	0.262
Sex (n, %)			0.001				0.004
Female	42 (33)	37 (59)			21 (40)	20 (77)	
Male	84 (67)	26 (41)			32 (60)	6 (23)	
Diabetes (n, %)			0.009				0.21
No	120 (95)	52 (83)			50 (94)	22 (85)	
Yes	6 (5)	11 (17)			3 (6)	4 (15)	
Hypertension (n, %)			0.54				0.106
No	85 (67)	46 (73)			34 (64)	22 (85)	
Yes	41 (33)	17 (27)			19 (36)	4 (15)	
Smoking (n, %)			0.111				0.275
No	80 (63)	48 (76)			35 (66)	21 (81)	
Yes	46 (37)	15 (24)			18 (34)	5 (19)	
Drinking (n, %)			0.365				0.551
No	93 (74)	51 (81)			38 (72)	21 (81)	
Yes	33 (26)	12 (19)			15 (28)	5 (19)	
Fever (n, %)			< 0.001				0.007
No	108 (86)	40 (63)			49 (92)	17 (65)	
Yes	18 (14)	23 (37)			4 (8)	9 (35)	
History of ipsilateral stone surgery (n, %)			0.058				0.631
No	89 (71)	35 (56)			37 (70)	16 (62)	
Yes	37 (29)	28 (44)			16 (30)	10 (38)	
Renal colic (n, %)			< 0.001				< 0.001
No	56 (44)	55 (87)			18 (34)	23 (88)	
Yes	70 (56)	8 (13)		35 (66)		3 (12)	

### Blood cells and infection characteristics

Table 2 shows the statistical differences in WBC (*p* = 0.001), neutrophils (*p* < 0.001), lymphocytes (*p* < 0.001), RBC (*p* < 0.001), hemoglobin (*p* < 0.001), CRP (*p* < 0.001), PCT (*p* < 0.001), and IL-6 (*p* < 0.001) between the two groups in terms of blood cell and infection analysis. With the exception of lymphocytes (*p* = 0.136), there were significant differences in the test set.

**Table 2:** Comparison results of blood cell and infection characteristics on two groups

Characteristic	Training set (*n* = 189)				Testing set (*n* = 79)		
	Hydronephrosis (*n* = 126)		Pyonephrosis (*n* = 63)	*p*	Hydronephrosis (*n* = 126)	Pyonephrosis (*n* = 63)	*p*
WBC (*10^9/L) (IQR)	7.36 (6.16, 9.07)		8.85 (7.24, 11.2)	0.001	7.22 (5.48, 9.3)	9.41 (6.72, 12.74)	0.013
Neutrophils (*10^9/L) (IQR)	4.63 (3.49, 6.35)		6.58 (4.99, 9.43)	< 0.001	4.6 (3.16, 6.35)	7.18 (4.39, 10.25)	0.004
Lymphocytes (*10^9/L)	1.87 ± 0.7		1.47 ± 0.63	< 0.001	1.6 (1.43, 2.18)	1.4 (1.27, 2)	0.136
N/L (IQR)	2.32 (1.7, 3.91)		4.71 (2.84, 7.96)	< 0.001	2.35 (1.87, 3.72)	5.33 (3.27, 7.85)	< 0.001
RBC (*10^9/L)	4.66 ± 0.77		4.16 ± 0.63	< 0.001	4.53 ± 0.67	4.11 ± 0.76	0.02
HGB (*10^9/L)	141.15 ± 23.3		123.08 ± 17.94	< 0.001	137.4 ± 21.94	117.92 ± 22.62	< 0.001
CRP (mg/L) (IQR)	7.78 (4.44, 22.11)		92.82 (54.44, 30.98)	< 0.001	9.53 (4.98, 40.76)	95.41 (56.53, 54.69)	< 0.001
PCT (ng/L) (IQR)	0.07 (0.04, 0.36)		1.4 (0.36, 6.86)	< 0.001	0.09 (0.06, 0.19)	0.62 (0.29, 4.43)	< 0.001
IL-6 (Pg/ml) (IQR)	8.37 (3.92, 22.91)		51.28 (28.91, 20.65)	< 0.001	7.56 (4.39, 33.95)	90.93 (24.58, 31.83)	< 0.001

### Blood biochemical characteristics

Table 3 illustrates the blood biochemistry variations between two groups in terms of uric acid (*p* = 0.02), albumin (*p* < 0.001), globulin (*p* < 0.001), G/A (*p* < 0.001), blood glucose (*p* < 0.001), and cholesterol (*p* < 0.001). The rest was consistent with the training set, and there was no significant change in cholesterol (*p* = 0.102) between the testing and training sets.

**Table 3:** Comparison results of blood biochemistry characteristics on two groups

Characteristic	Training set(*n* = 189)			Testing set(*n* = 79)		
	Hydronephrosis (*n* = 126)	Pyonephrosis (*n* = 63)	*p*	Hydronephrosis (*n* = 126)	Pyonephrosis (*n* = 63)	*p*
Urea (umol/L) (IQR)	6.4 (5.04, 8.77)	6.31 (4.38, 9.01)	0.369	7.46 (5.7, 10.08)	6.42 (4.8, 8.93)	0.251
Uric acid (umol/L) (IQR)	410.8 (318, 482)	340 (279, 443.5)	0.02	455 (345, 542)	347.5 (298.8, 427.25)	0.014
Albumin (g/L) (IQR)	40.65 (37.02, 43.65)	36.3 (32.7, 38.65)	< 0.001	41.4 (37.8, 43.2)	36 (32.38, 40.12)	< 0.001
Globulin (g/L)	31.06 ± 5.55	35.55 ± 5.9	< 0.001	31.3 (29.2, 33.7)	38.35 (33.62, 42.62)	< 0.001
G/A (IQR)	1.31 (1.09, 1.48)	0.96 (0.88, 1.19)	< 0.001	1.3 ± 0.26	0.99 ± 0.27	< 0.001
Blood glucose (mmol/L) (IQR)	5.04 (4.64, 5.74)	5.81 (5.08, 7.73)	< 0.001	5.3 (4.68, 5.8)	6 (5.11, 7)	0.008
Cholesterol (mmol/L)	4.64 ± 0.95	4.08 ± 0.83	< 0.001	4.45 ± 0.87	4.08 ± 0.94	0.102
Triglyceride (mmol/L) (IQR)	1.56 (1.08, 2.01)	1.49 (1.12, 1.88)	0.689	1.47 (1.13, 2.25)	1.42 (1.18, 1.92)	0.456

### Urine characteristics

Urine analytical results are displayed in Table 4. In the testing set, urine WBC (*p* < 0.001), urine culture (*p* = 0.017), and urine nitrite (*p* = 0.034) were statistically different from two groups, but only urine WBC (*p* = 0.01) were in the training set.

**Table 4:** Comparative results of urine characteristics on two groups

Characteristic	Training set(*n* = 189)			Testing set(*n* = 79)		
	Hydronephrosis (*n* = 126)	Pyonephrosis (*n* = 63)	*p*	Hydronephrosis (*n* = 126)	Pyonephrosis (*n* = 63)	*p*
PH (IQR)	6 (6, 6.5)	6 (6, 6.5)	0.948	6 (5.8, 6.5)	6.25 (5.5, 6.5)	0.525
Urine RBC (/ul) (IQR)	33.27 (11.12, 105.6)	54.74 (15.8, 211.2)	0.368	25.4 (11.2, 177.22)	38.75 (12.53, 91.95)	0.835
Urine WBC (/ul) (IQR)	77.1 (5, 500)	500 (4, 1502.86)	0.01	58 (4, 440.7)	500 (65.7, 1444.75)	0.004
Urine ulture (n, %)			0.097			0.017
Negative	96 (76)	40 (63)		42 (79)	13 (50)	
Positive	30 (24)	23 (37)		11 (21)	13 (50)	
Nitrite (n, %)			0.947			0.034
Negative	104 (83)	51 (81)		49 (92)	19 (73)	
Positive	22 (17)	12 (19)		4 (8)	7 (27)	
Urinary bacterial count (CFU/mL) (IQR)	67.35 (30.4, 243.72)	205.8 (28.7, 3849.6)	0.019	71.2 (37.84, 187.1)	160.85 (70.93, 417.2)	0.015

### Stone-related characteristics

Table 5 shows the pertinent stone properties. In the training set, there were differences between two groups in terms of the maximal cross-sectional area (*p* = 0.006), stone position (*p* < 0.001), stone number (*p* = 0.036), and HU value of effusion (*p* < 0.001). In the testing set, there were additional variations in staghorn calculi (*p* = 0.004) and stone density (*p* = 0.005).

**Table 5:** Comparative results on stone-related characteristics on two groups

Characteristic	Training set (*n* = 189)			Testing set (*n* = 79)		
	Hydronephrosis (*n* = 126)	Pyonephrosis (*n* = 63)	*p*	Hydronephrosis (*n* = 126)	Pyonephrosis (*n* = 63)	*p*
Maximal cross-sectional area (mm2) (IQR)	112 (48, 241.25)	200 (96, 332.5)	0.006	88 (42, 153)	247.5 (140.75, 447)	< 0.001
Density (HU) (IQR)	860 (589.25, 1098.62)	863 (654.5, 1080.5)	0.739	709.07 ± 360.45	935.27 ± 299.75	0.005
Side (n, %)			0.319			0.33
Left	47 (37)	29 (46)		26 (49)	9 (35)	
Right	79 (63)	34 (54)		27 (51)	17 (65)	
Position (n, %)			< 0.001			0.009
Renal	51 (40)	29 (46)		20 (38)	13 (50)	
Ureter	58 (46)	10 (16)		29 (55)	6 (23)	
Renal + Ureter	17 (13)	24 (38)		4 (8)	7 (27)	
Number (n, %)			0.036			0.035
Single	32 (25)	7 (11)		17 (32)	2 (8)	
Multiple	94 (75)	56 (89)		36 (68)	24 (92)	
Staghorn (n, %)			0.054			0.004
No	117 (93)	52 (83)		52 (98)	20 (77)	
Yes	9 (7)	11 (17)		1 (2)	6 (23)	
HU value of effusion	4 (-1.98, 7.47)	7.7 (6, 13.05)	< 0.001	2.44 ± 7.05	10.1 ± 7.78	< 0.001

### Five ML prediction models are evaluated

Table 6 shows that the training dataset’s greatest AUC was generated by RF (AUC 1.000, 95%*CI* 0.999-1.000), followed by XGBoost (AUC 0.999, 95%*CI* 0.982-1.000), GBDT (AUC 0.977, 95%*CI* 0.952-1.000) and SVM (AUC 0.971, 95%*CI* 0.946-0.996). The lowest AUC (AUC 0.938, 95%*CI* 0.899-0.977) was found utilizing LR. The five prediction models performed satisfactorily in terms of prediction on the training set; the AUC of RF was 1.000, while the AUC of the other models was greater than 0.900, all falling within the 95%*CI*.

GBDT (AUC 0.967, 95%*CI* 0.935-1.000) had the greatest AUC in the test set. LR had the next highest AUC (AUC 0.957, 95%*CI* 0.911-1.000), followed by XGBoost (AUC 0.950, 95%*CI* 0.901-0.990), SVM (AUC 0.939, 95%*CI* 0.889-0.989) and RF (AUC 0.924, 95%*CI* 0.859-0.988).

LR, GBDT and RF models have the best accuracy, followed by SVM, while XGBoost (0.873) with the lowest accuracy. At 0.923 and 0.887, respectively, the LR model’s sensitivity and specificity were the greatest among the five models.

**Table 6:** Five ML prediction models’ outcomes

Model	AUC	Accuracy	Sensitivity	Specificity	95%*CI*
XGBoost	0.950	0.850	0.880	0.800	0.901, 0.990
RF	0.924	0.873	0.891	0.833	0.859, 0.988
SVM	0.939	0.860	0.889	0.800	0.889, 0.989
GBDT	0.967	0.873	0.891	0.833	0.935, 1.000
LR	0.957	0.873	0.923	0.887	0.911, 1.000

**Fig. 1:**
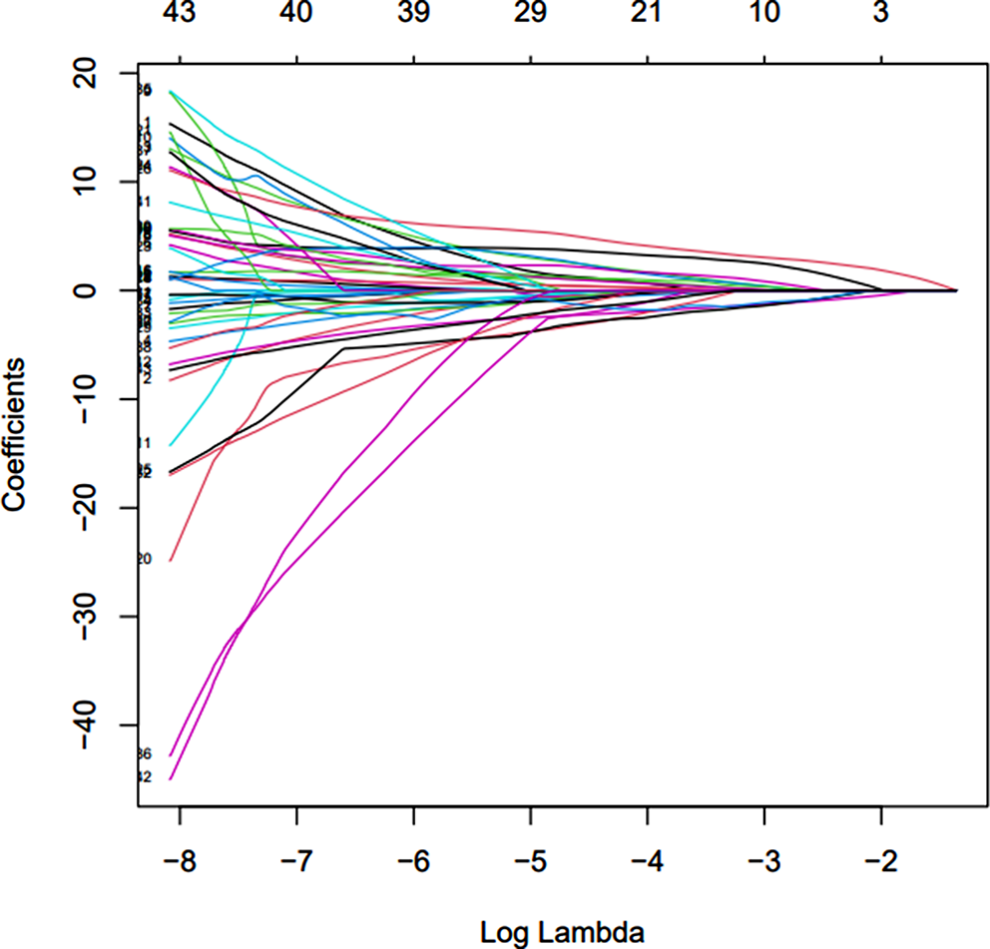
Lasso regression coefficient and log (λ) value

Pyonephrosis was identified as the cause feature in the training set of 189 patients. The collinearity feature was eliminated using the Lasso regression and characteristic screening was carried out (Fig. 1).

**Fig. 2:**
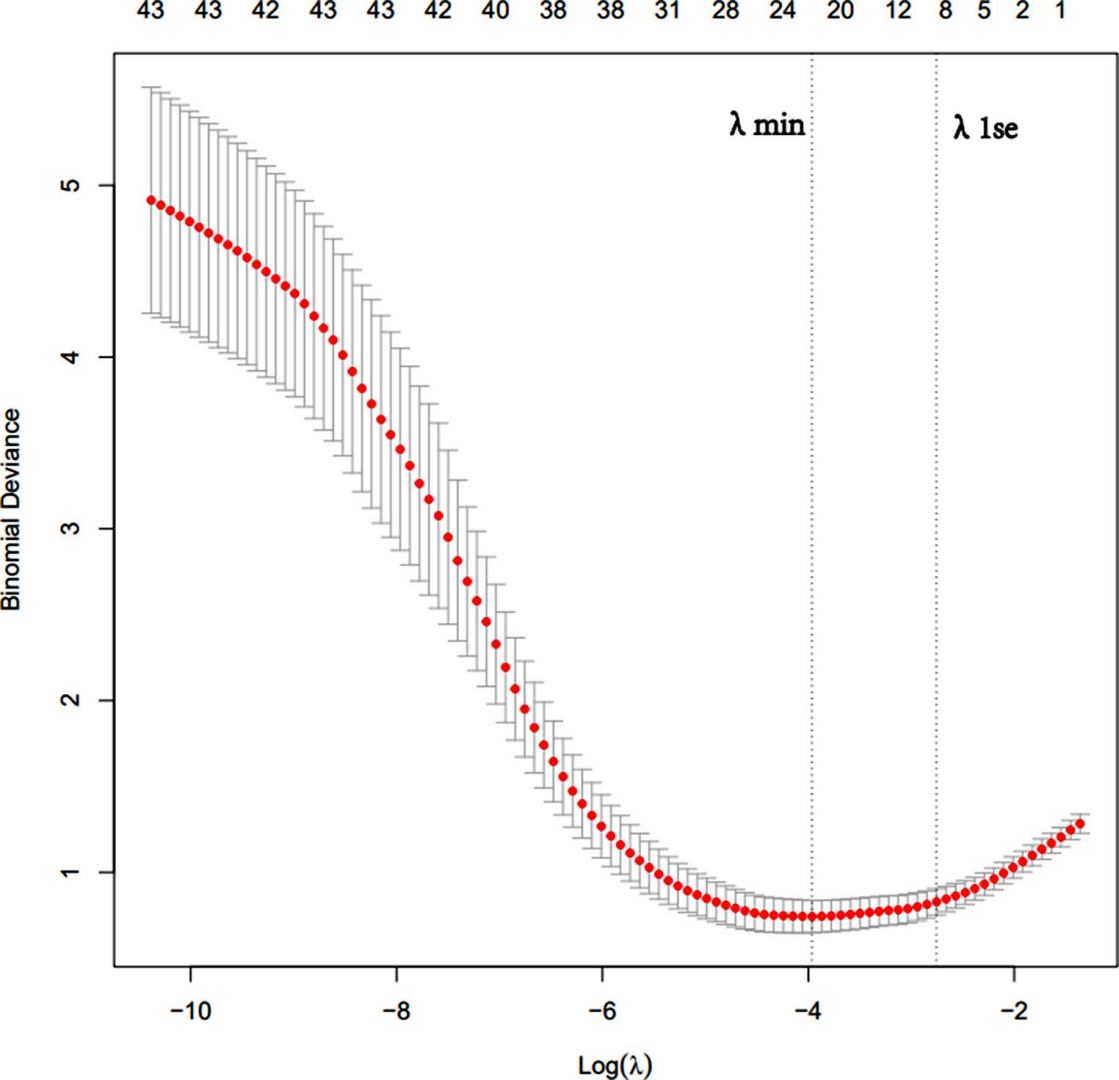
Log (λ) value and model error

The λmin that the model error is the lowest and the λ1se that within a standard error range are shown (Fig. 2). The fitting impact of the model is better the narrower the ordinate axis (degree of freedom), according to Lasso regression analysis on a 10-fold cross-validation curve.

In this research, the binary logistic regression model was established by selecting the appropriate characteristics of λmin and λ1se. We discovered that on both the training and testing sets, the LR models’ AUC were same, thus chose nine features that corresponded to the λ1se. Nine characteristics were determined using Lasso regression: globulin, G/A, diabetes, renal colic, hemoglobin, CRP, IL-6, urine bacterial count and HU value of effusion.

The nine characteristics that Lasso regression assessed are first subjected to single-factor and multi-factor LR analysis, as indicated in Table 7. Five characteristics have been demonstrated to be independent risk factors by multi-factor LR, while the single-factor LR analysis of the nine features revealed significant differences in both the hydronephrosis group and the pyonephrosis group, with *P* < 0.05 serving as the screening criterion.

Diabetes (*OR* = 16.32, 95%*CI* 2.02-131.67, *p* = 0.009), renal colic (*OR* = 0.06, 95%*CI* 0.02-0.05, *p* < 0.001), HU value of effusion (*OR* = 1.14, 95%*CI* 1.06-1.23, *p* < 0.001), hemoglobin (*OR* = 0.97, 95%*CI* 0.95-1.00, *p* = 0.026) and CRP (*OR* = 1.02, 95%*CI* 1.01-1.04, *p* < 0.001) were the five independent risk factors.

Given the regression coefficients of the characteristics found in Table 8, the following formula is used to computing our LR model: logit (Y) = 3.525–1.532 * diabetes-3.456 * renal colic + 0.178 * HU value of effusion − 0.046 * hemoglobin + 0.030 * CRP. The binary predictive features in the algorithm are evaluated as 0 or 1.

**Table 7:** Single and multiple factors LR results of nine characteristics

	Single factors LR		Multiple factors LR	
Characteristic	*OR (*95%*CI)*	*p*	*OR* (95%*CI*)	*p*
Diabetes	4.23 (1.49–12.05)	0.007	16.32 (2.02-131.67)	0.009
Renal colic	0.12 (0.05–0.26,)	< 0.001	0.06 (0.02–0.25)	< 0.001
HU value of effusion	1.10 (1.05–1.16)	< 0.001	1.14 (1.06–1.23)	< 0.001
Hemoglobin	0.96 (0.95–0.98)	< 0.001	0.97 (0.95-1.00)	0.026
CRP	1.02 (1.02–1.03)	< 0.001	1.02 (1.01–1.04)	< 0.001
IL-6	1.01 (1.01–1.02)	< 0.001	1.00 (1.00-1.01)	0.386
Urine bacterial count	1.00 (1.00–1.00)	0.005	1.00 (1.00–1.00)	0.053
Globulin	1.15 (1.08–1.22)	< 0.001	1.04 (0.92–1.17)	0.558
G/A	0.01 (0.00-0.05)	< 0.001	0.64 (0.03–16.30)	0.787

**Table 8:** Five characteristics of the LR model

	Coefficient	Standard error	Z-statistics	*p* (>|Z|)
Intercept	3.5247	2.6130	1.35	0.1774
Diabetes	-1.5315	1.5335	-1.00	0.3179
Renal colic	-3.4561	1.1287	-3.06	0.0022
HU value of effusion	0.1778	0.0590	3.01	0.0026
Hemoglobin	-0.0463	0.0210	-2.20	0.0276
CRP	0.0296	0.0092	3.21	0.0013

**Fig. 3:**
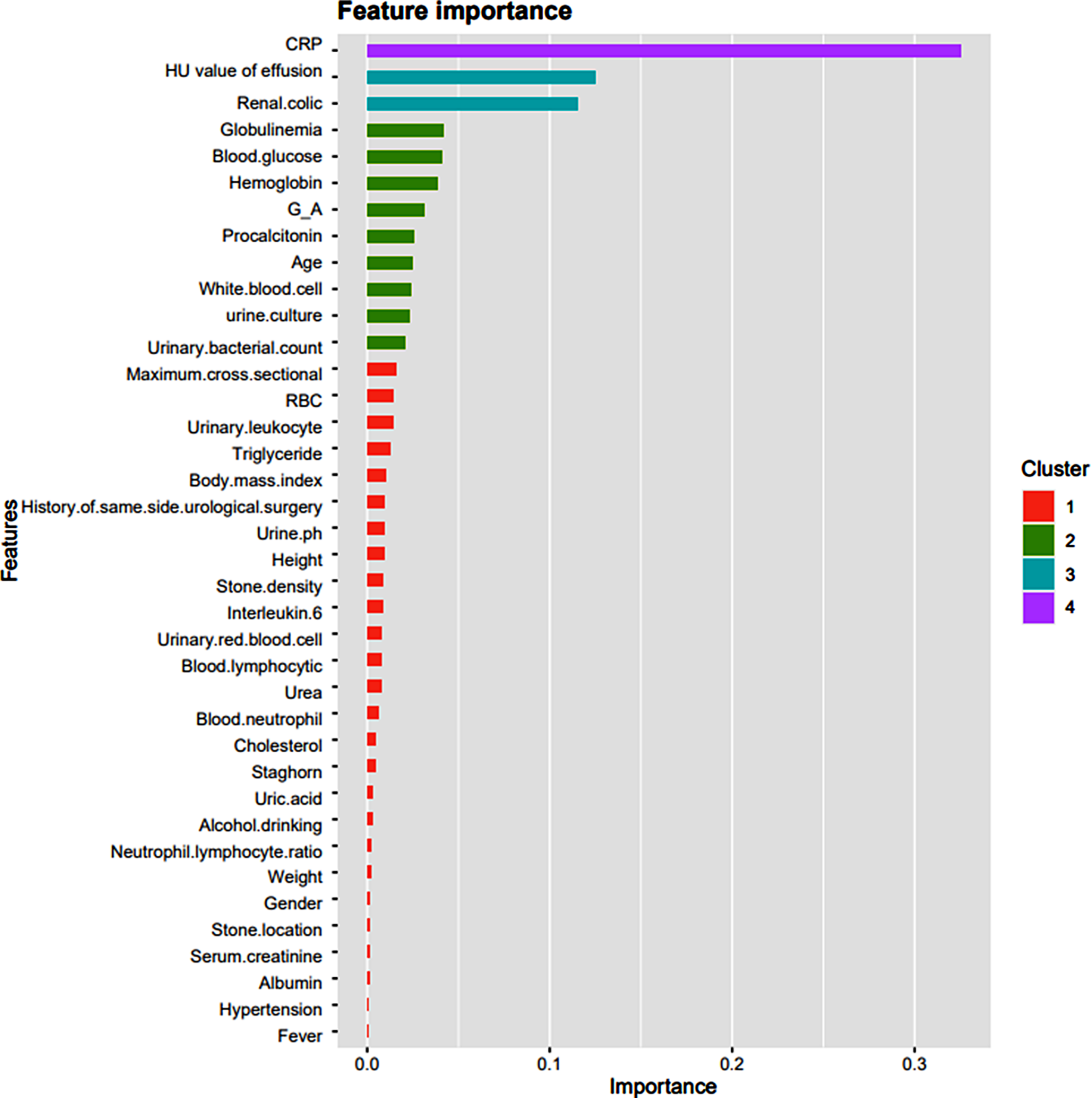
The characteristics importance ranking of XGBoost model

**Fig. 4:**
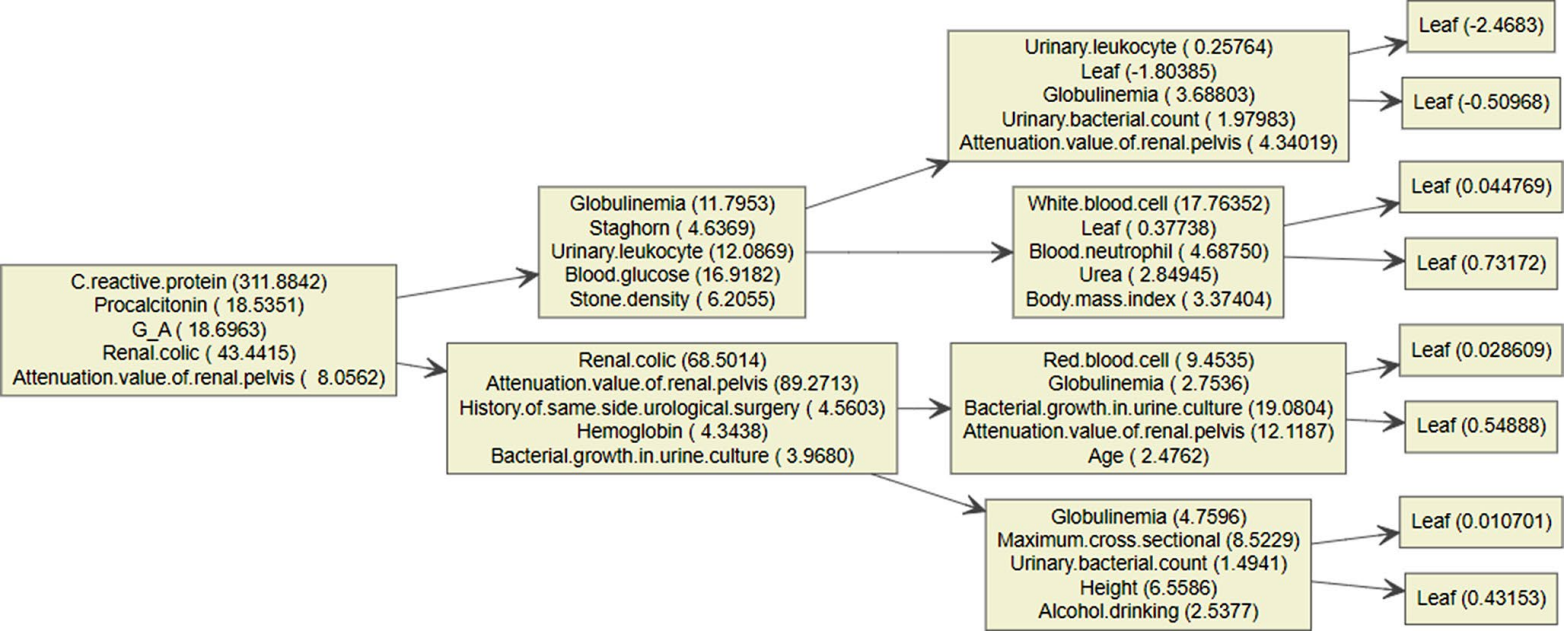
Multi classification tree set of XGBoost model

Gradient boosting tree is the foundation upon which the XGBoost model is constructed. The contribution of the chosen feature is the gain on each node. As seen in Fig. 3, we total up each feature’s contribution to determine the characteristic’s relevance rating. We grouped the characteristics based on their relative value. Figure 4 shows the collection of a few trees in the model. The foremost clinical characteristic in the XGBoost model is CRP, which is followed by hemoglobin, blood glucose, renal colic, globulin and HU value of effusion. The XGBoost prediction model’s accuracy was 0.968, its sensitivity and specificity were 0.962 and 0.981, respectively, and its AUC on the training set was 0.990 (95%*CI*, 0.982-1.000).

.

**Fig. 5:**
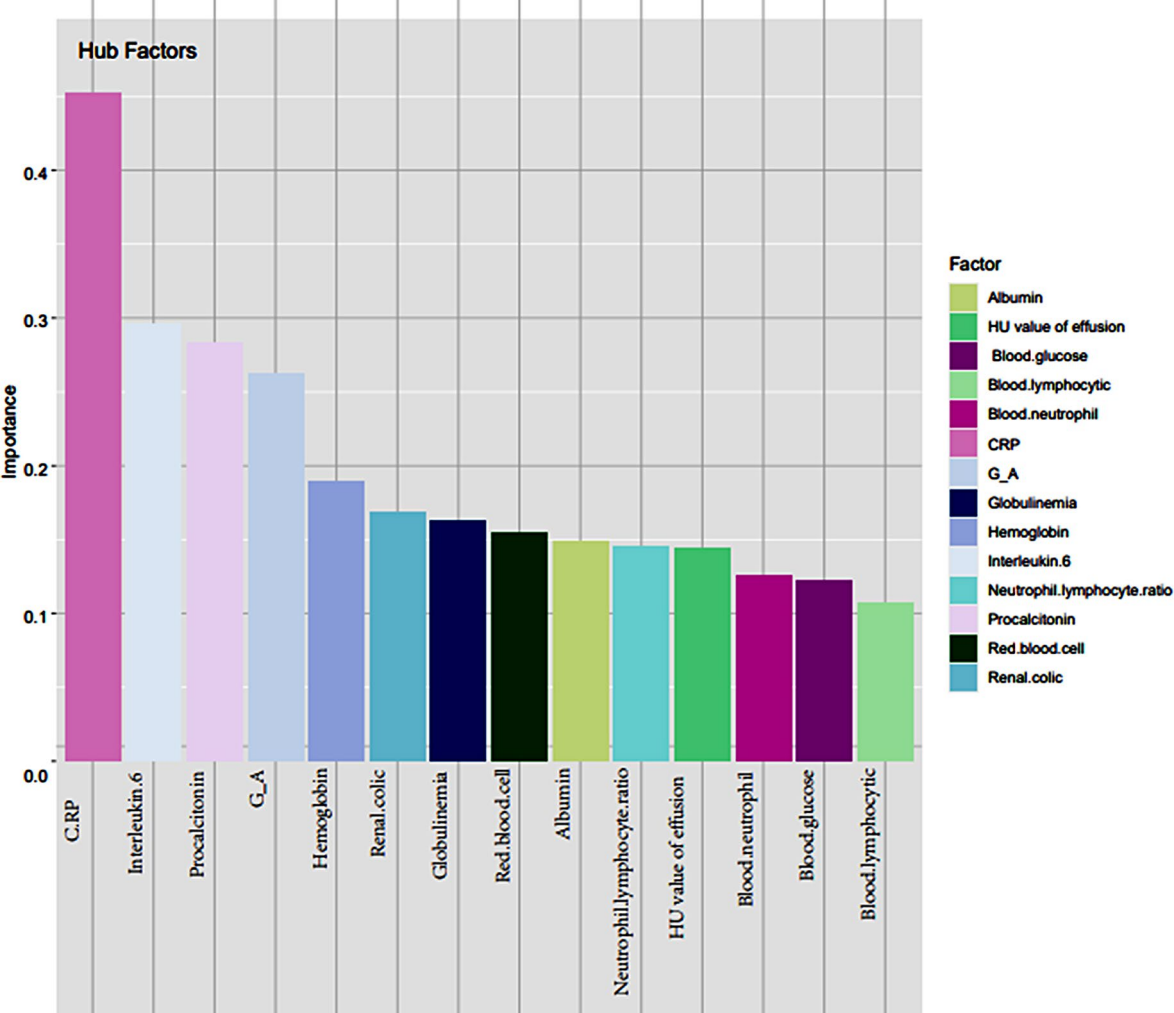
The SVM model’s significance is one of the top 14 characteristics

An approach to sort characteristics using the SVM algorithm is to add hierarchical characteristics one at a time. The top 14 characteristics are arranged as shown (Fig. 5): N/L, HU value of effusion, hemoglobin, renal colic, globulin, WBC, albumin, PCT, IL-6 and blood neutrophils.

**Fig. 6:**
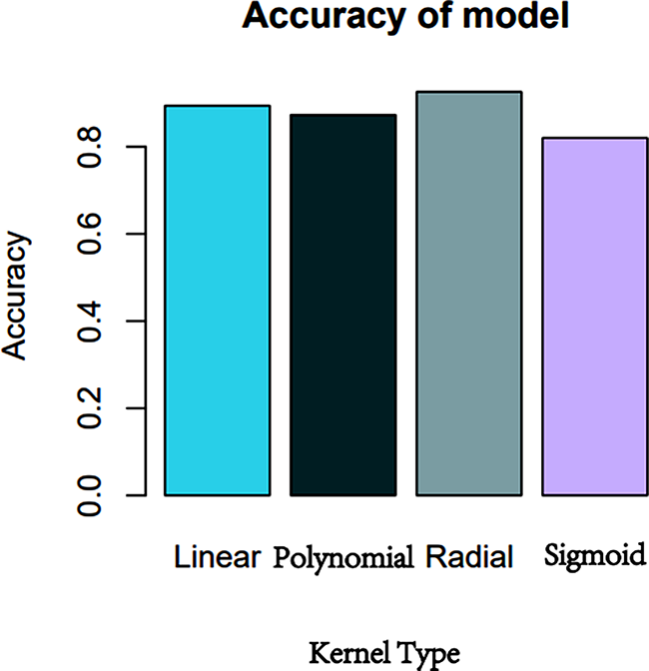
Accuracy of four SVM kernel functions predictions

Figure 6 shows the prediction accuracy of the four linear kernel functions of SVM: linear, polynomial, radial and sigmoid. The results are 0.894, 0.873, 0.911, and 0.820, respectively. In constructing the SVM model, we selected the most accurate Radial Kernel. AUC of the SVM model was 0.971 (95%*Cl*, 0.946-0.996) on the training set, with accuracy of 0.947, sensitivity of 0.968, and specificity of 0.908. On the testing set, AUC was 0.939 (95%*Cl*, 0.889-0.989), with accuracy of 0.860, sensitivity of 0.889, and specificity of 0.800.

**Fig. 7:**
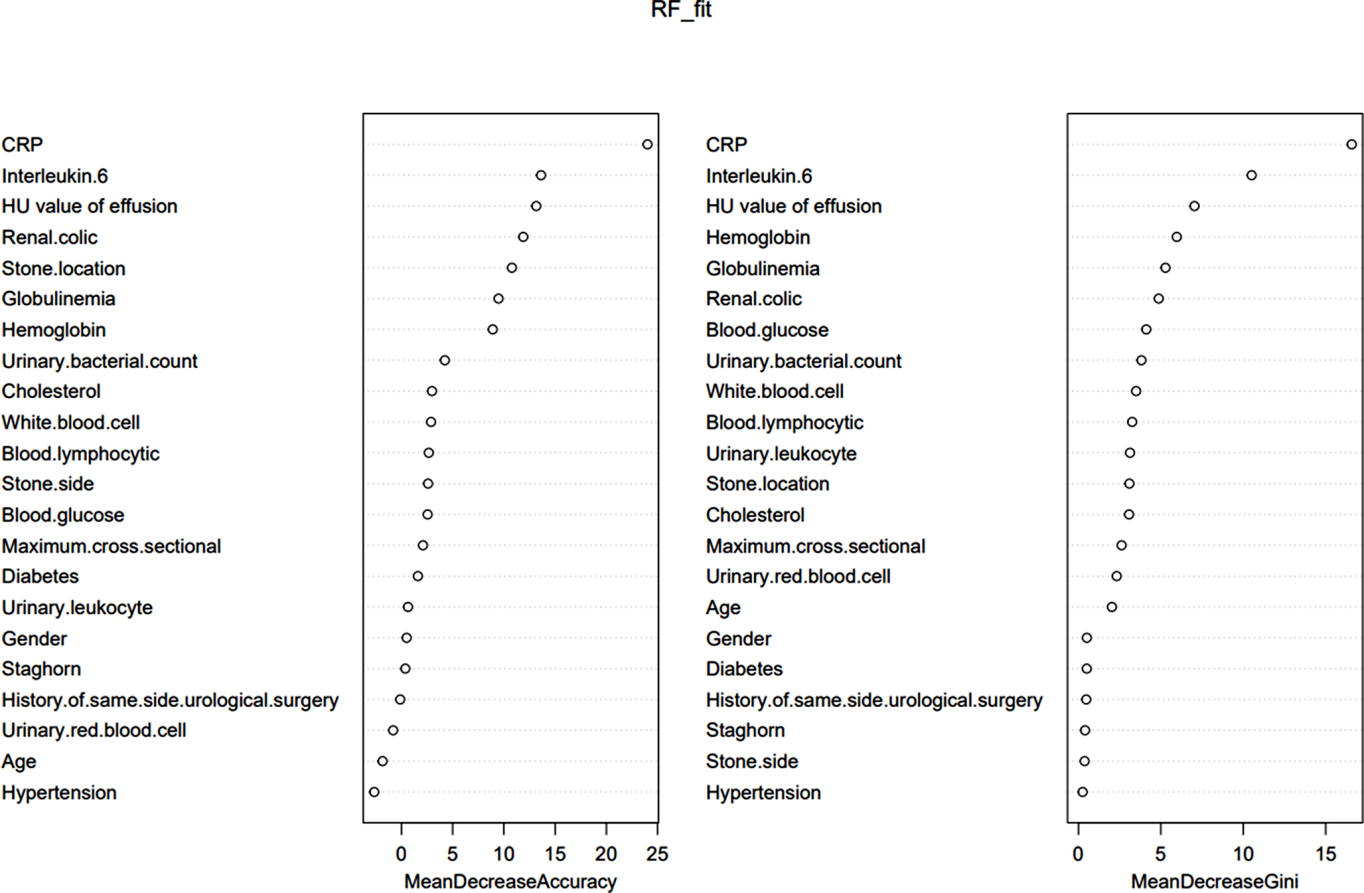
The characteristics importance ranking of RF model

Many decision trees compose RF. Every decision tree is a separate entity. When we combined them, receiving a prediction result that is derived from the weighted average of all the trees’ predictions. Mean decrease gini (MDG) and Mean decrease accuracy (MDA) are used to rank the significance of each characteristic, the greater the value, the more significant the trait (Fig. 7).

CRP (23.801), interleukin 6 (14.099), renal colic (13.762), HU value of effusion (11.855), stone position (9.366), and globulin (8.880) were the attributes of MDA > 5. CRP (17.353), interleukin 6 (10.500), HU value of effusion (7.168), globulin (5.329), and hemoglobin (5.305) were the top five most significant predictors in MDG. Overall, MDA and MDG feature significance ranking findings are comparable.

To construct the RF model, we utilized 158 trees that have the lowest overall error rate. For the model training set, the AUC was 1.000 (95%*Cl*, 0.999-1.000), accuracy, sensitivity and specificity were 1.000. The AUC of the testing was 0.924 (95%*Cl*, 0.859-0.988), the accuracy was 0.873, the sensitivity was 0.891, and the specificity was 0.833.

**Fig. 8:**
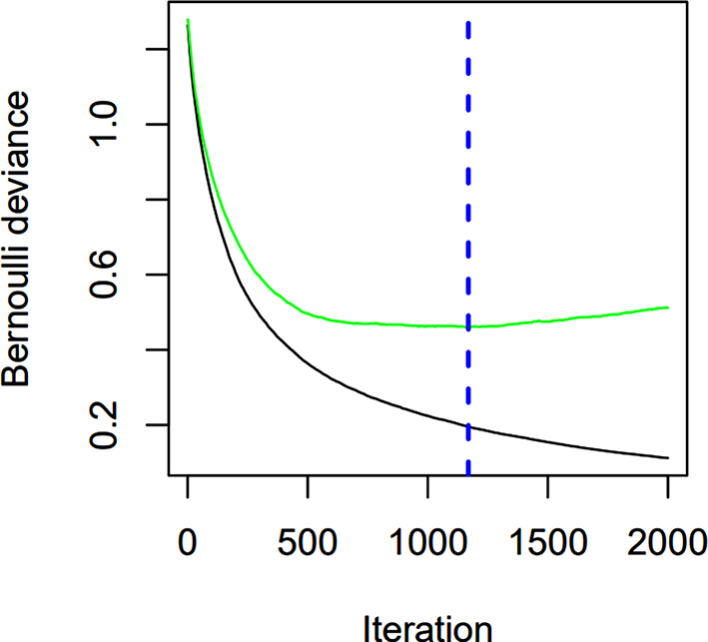
The ideal number of GBDT model iterations

The decision tree model is iteratively constructed with GBDT utilizing the gradient boosting technique. In an effort to lower the prediction error of the current model on the training data, the model will train a new decision tree in each iteration using the residual of the previous model. we reached at the perfect number of model iterations is 1169 using 10-fold cross-validation (Fig. 8).

**Fig. 9:**
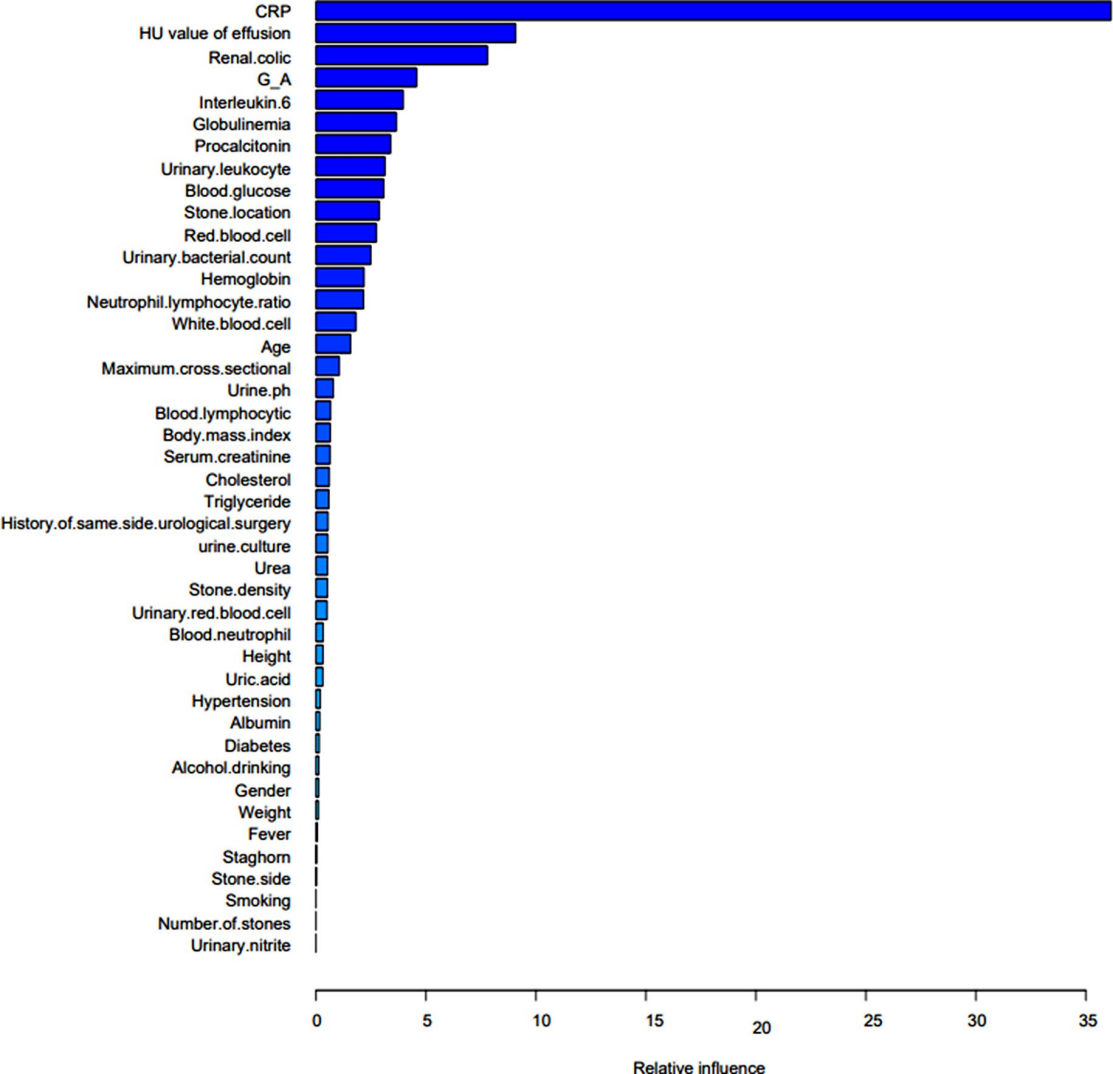
The characteristics importance ranking of GBDT model

The characteristics of the GBDT model are arranged based on their relative significance. CRP remains the most crucial clinical characteristic, followed by renal colic, HU value of effusion, G/A, IL-6, globulin, and PCT (Fig. 9). The AUC of the GBDT model training set was 0.977 (95%*Cl*, 0.952-1.000), the accuracy was 0.952, the sensitivity was 0.961, and the specificity was 0.935. In the testing, the accuracy, sensitivity, specificity and AUC were 0.873, 0.891 and 0.833 (95%*Cl*, 0.935-1.000).

**Fig. 10:**
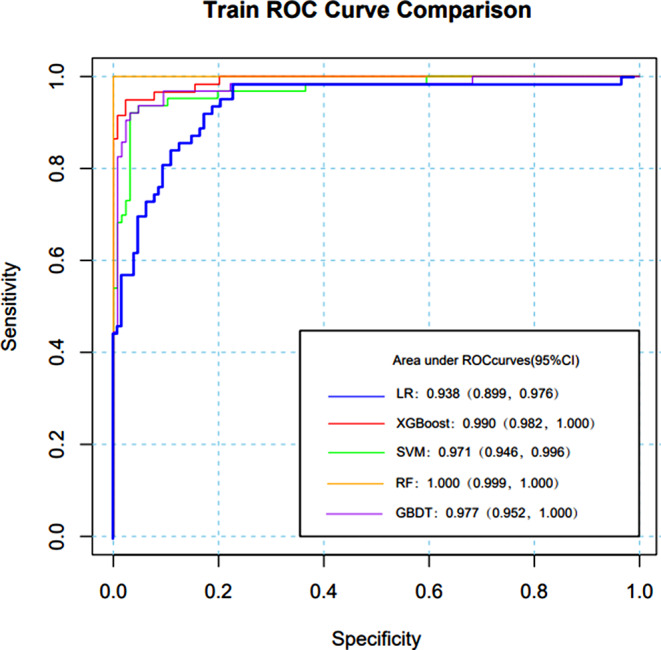
ROC of five prediction models for ML on the training set

The models with the greatest AUC were RF (AUC 1.000, 95%*CI* 0.999-1.000) (Fig. 10), followed by XGBoost (AUC 0.999, 95%*CI* 0.982-1.000), GBDT (AUC 0.977, 95%*CI* 0.952-1.000) and SVM (AUC 0.971, 95%*CI* 0.946-0.996). The lowest AUC was found with LR (AUC 0.938, 95%*CI* 0.899-0.977). All five of the prediction models performed well in terms of prediction on the training set; the AUC of RF was 1.000, while other models were greater than 0.900, all falling within the 95%*CI*.

**Fig. 11:**
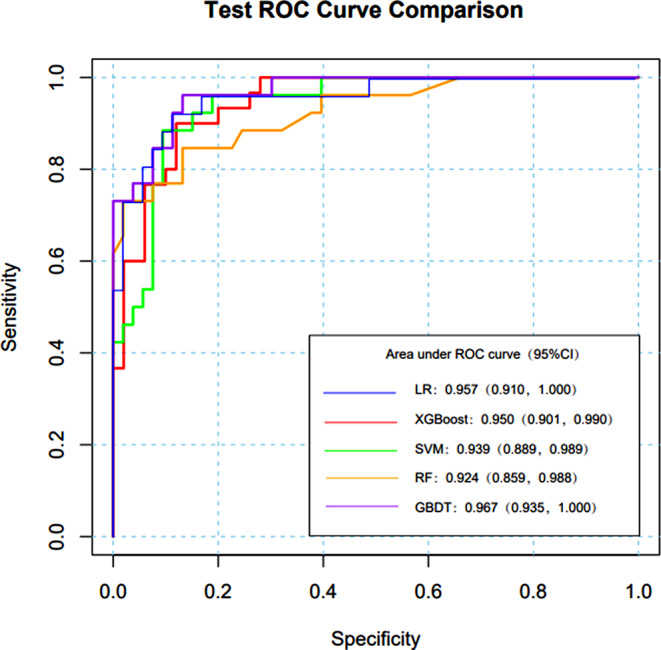
ROC of five prediction models for ML on the testing set

Among the models tested (Fig. 11), GBDT (AUC 0.967, 95%*CI* 0.935-1.000) had the greatest AUC, in the testing set followed by RF (AUC 0.924, 95%*CI* 0.859-0.988), XGBoost (AUC 0.950, 95%*CI* 0.901-0.990), SVM (AUC 0.939, 95%*CI* 0.889-0.989) and LR (AUC 0.957, 95%*CI* 0.911-1.000).

### Calibration analysis of LR model

**Fig. 12:**
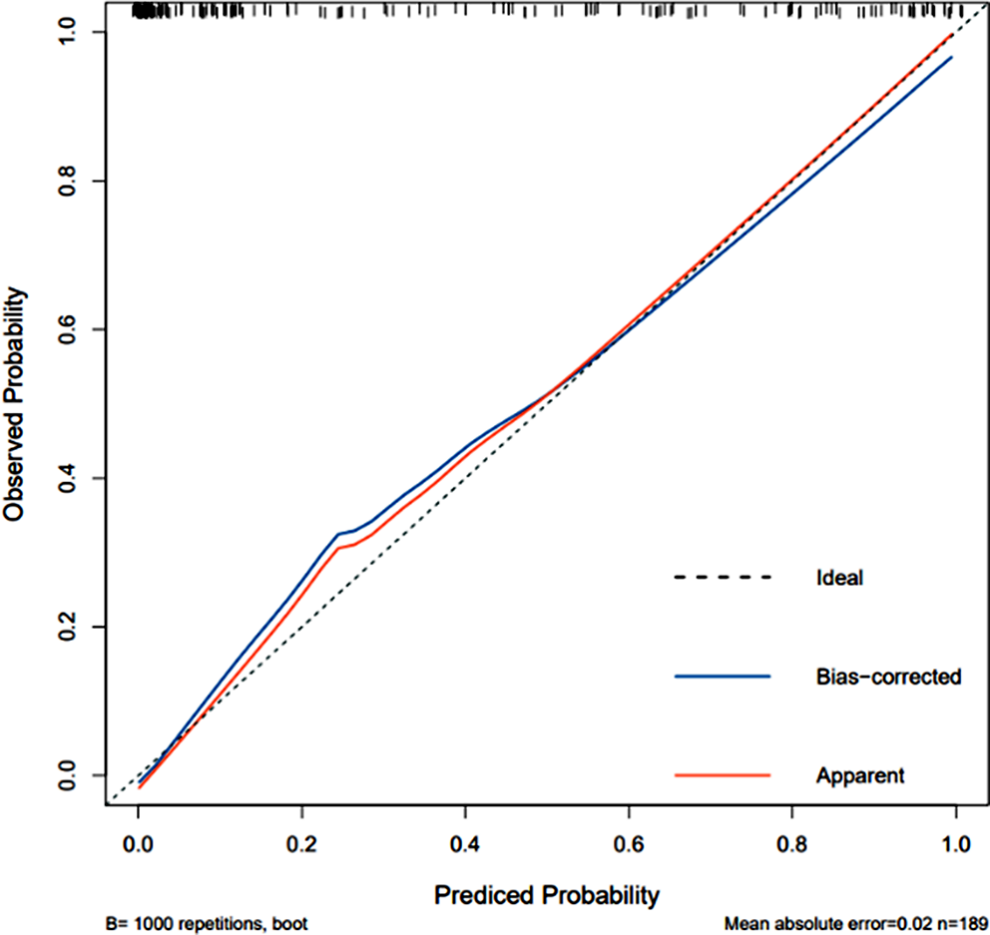
Calibration curve of LR model on training set

**Fig. 13:**
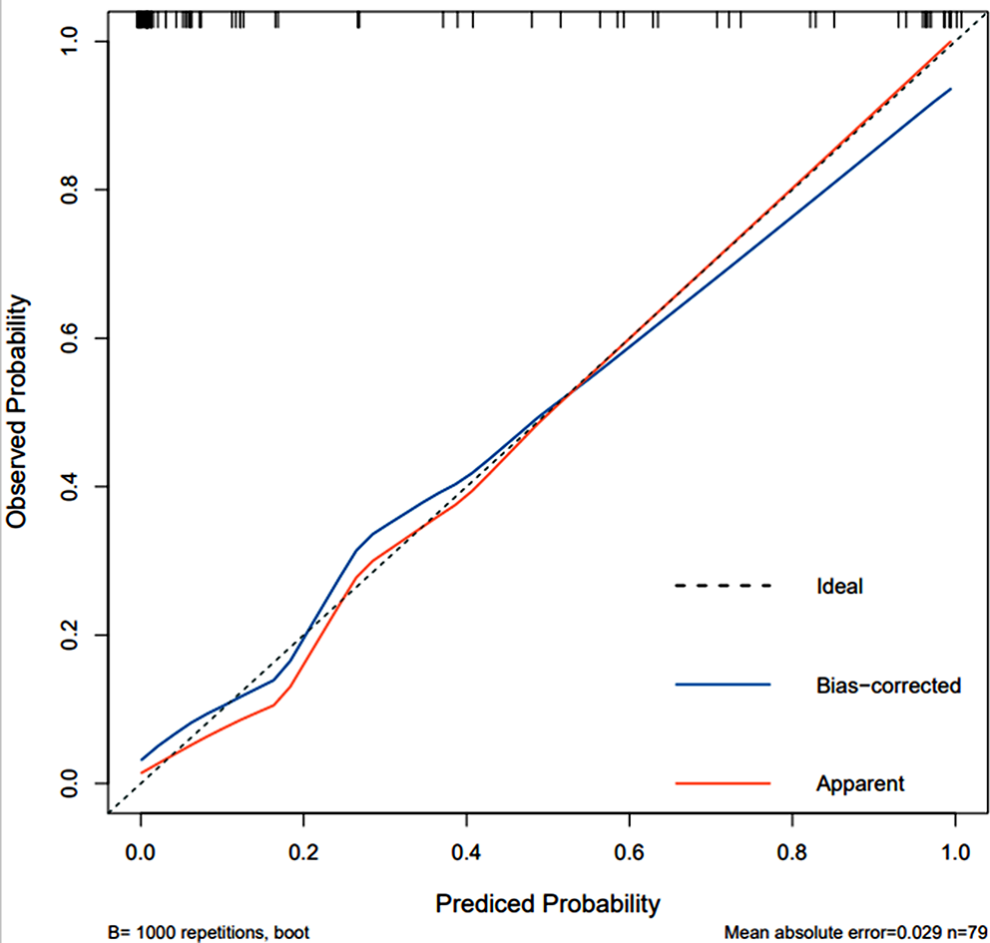
Calibration curve of LR model on testing set

In our study, the GBDT model had the greatest AUC (AUC 0.967, 95%*CI* 0.935-1.000), with an accuracy rate of 0.873, a sensitivity of 0.891 and a specificity of 0.833. Followed by the LR model (AUC 0.957, 95%*CI* 0.911-1.000), the sensitivity and specificity were 0.923 and 0.887. The LR model is regarded as the most effective model in this study because to its clinical interpretability and practicability. The model calibration of the training set and the testing set of the LR model were confirmed using the Bootstrap resampling approach (*n* = 1000) (Figs. 12 and 13).

### Clinical applicability analysis of LR model

**Fig. 14:**
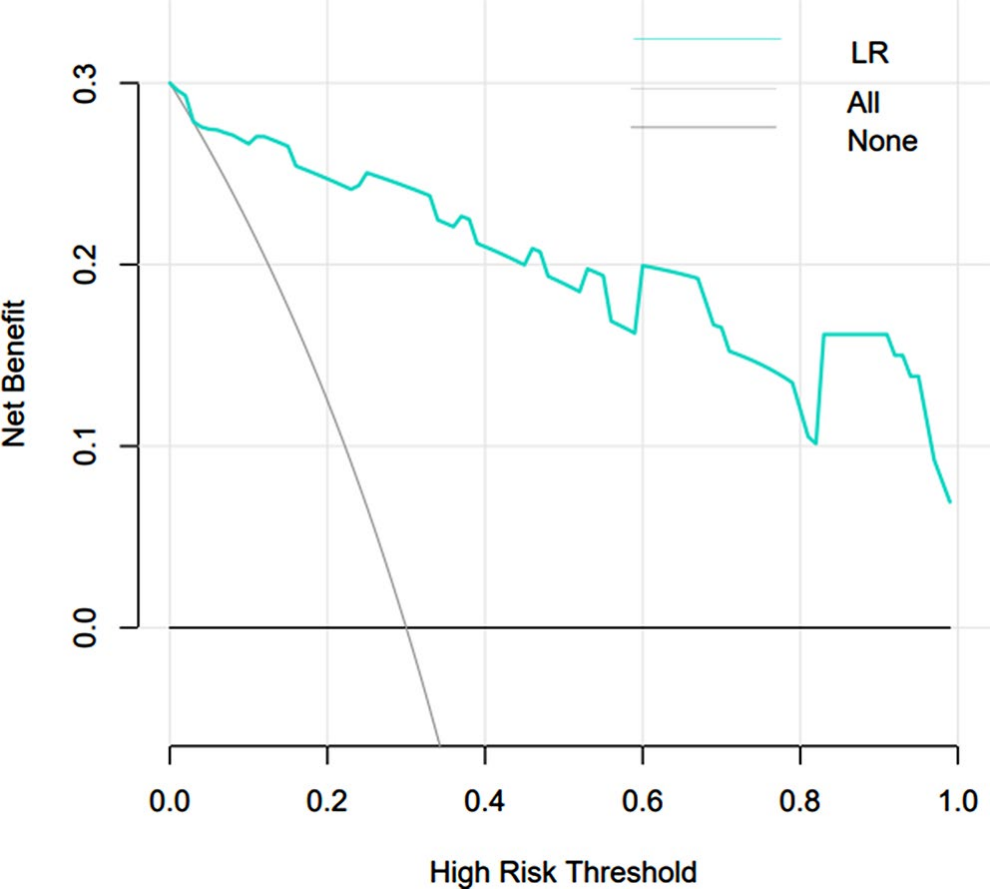
DCA curve of the LR model on the testing set

The DCA curve was generated for the LR model and its applicability was examined (Fig. 14) The two extreme curves in the testing set, All and None, are lower than DCA curve of the model. The results demonstrated that LR model offers a larger net benefit for therapeutic intervention for patients with pyonephrosis. All is the net income line when all patients were intervened. None is the net benefit line for all patients without intervention.

### Nomogram of LR model

**Fig. 15:**
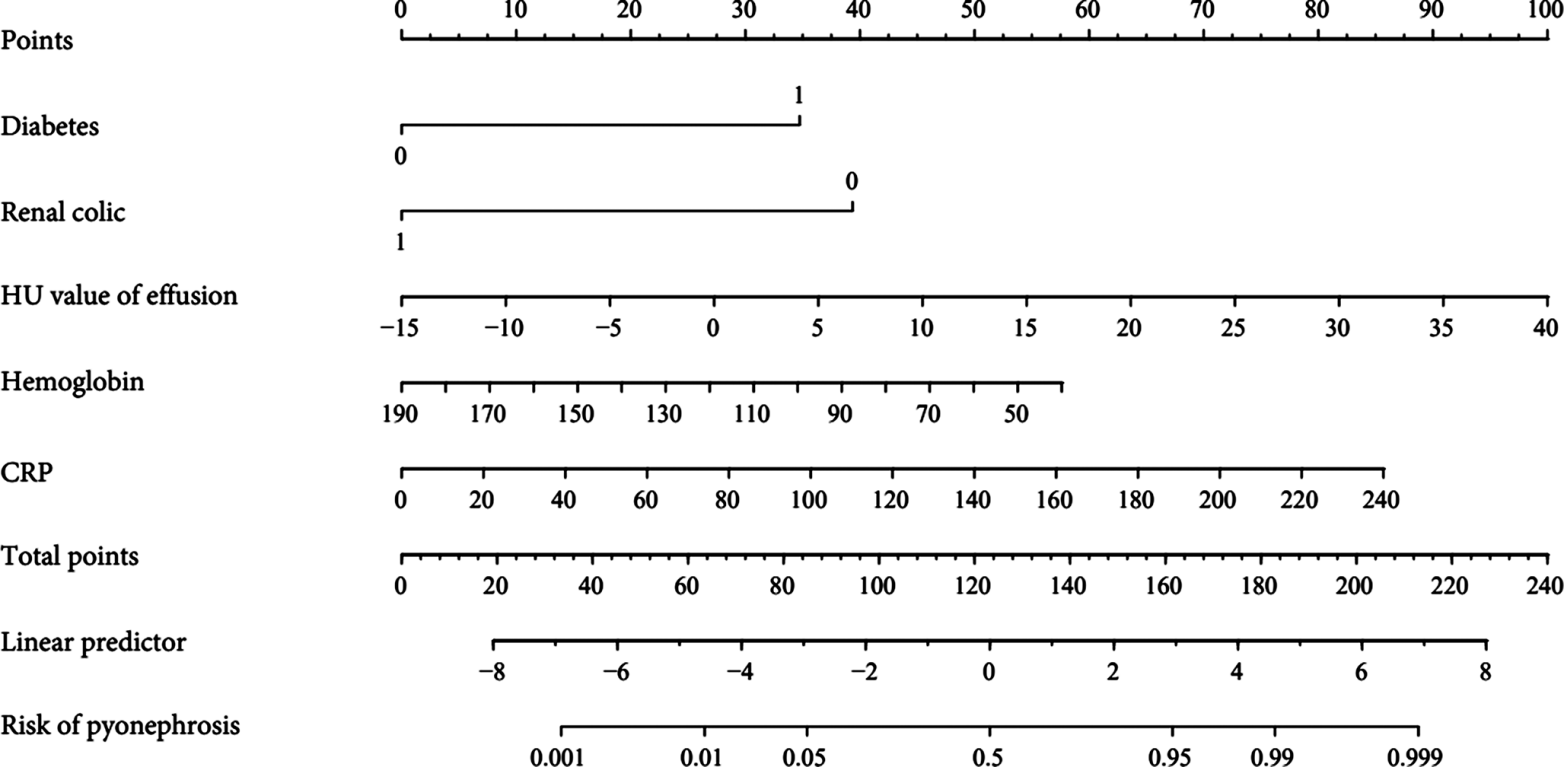
Nomogram of LR model

Nomogram integrated five elements of diabetes, renal colic, hemoglobin, CRP and HU value of effusion, then utilized a line segment with a scale to depict the LR model. It is convenient for urologists to obtain the probability of pyonephrosis by summing the scores corresponding to the five features (Fig. 15). It is drawn on the same plane in a specific proportion to express the relationship between the various characteristics in the prediction model.

## Discussion

Pyonephrosis is usually caused by infection after obstruction. Bacteria enter the kidney and cause infection of the renal parenchyma [29]. Multiple symptoms, including as fever, chills and low back discomfort, could come from this. Renal function may eventually be harmed by a persistent infection that negatively affects the nephron and renal tubules. Bacteria enters into the bloodstream as a consequence of acute renal empyema, leading to serious infectious disorders such sepsis that may harm the body functions [30].

While the patient’s symptoms, hematuria analysis results and imaging findings ought to all be taken into account in the diagnosis of pyonephrosis, while some patients with chronic pyonephrosis frequently have no symptoms at all, such as low back pain and a persistent fever. Additionally, there were no significant abnormalities in infection indicators like as white blood cells, neutrophils and positive urine culture. It is challenging to identify hydronephrosis complicated by infection via ultrasound or CT imaging, which results in missed diagnoses for those with this condition.

Numerous researchers have examined the variations in clinical information between patients with hydronephrosis and pyonephrosis in an attempt to identify the risk factors for the development of pyonephrosis. However, the studies vary in design and in the effectiveness of their diagnostic methods. We discovered that the following were independent risk factors for the development of calculous pyonephrosis: hemoglobin, renal colic, CRP, HU value of effusion and diabetes. Out of the five models, CRP is the most significant attribute. The top five characteristics in the four models of LR, XGBoost, RF, and GBDT include CRP, renal colic, and HU value of effusion.

### Examination of risk factors

#### Blood characteristics

WBC (*p* = 0.001), neutrophils (*p* < 0.001), lymphocytes (*p* < 0.001), RBC (*p* < 0.001), hemoglobin (*p* < 0.001), CRP (*p* < 0.001), PCT (*p* < 0.001), and IL-6 (*p* < 0.001) were significantly different between the hydronephrosis group and the pyonephrosis group in the single factor analysis of blood cell analysis and infection. The findings of the multivariate LR analysis showed that hemoglobin and CRP were the only two independent risk variables for the incidence of pyonephrosis, there was no collinearity in the occurrence of pyonephrosis.

The body releases CRP, a plasma protein, which rises dramatically in response to inflammation or tissue injury. Physicians can monitor disease activity, gauge the degree of inflammation, and inform treatment plan development by measuring CRP, which is often employed as a biomarker of clinical inflammation and infection [31]. This inflammatory marker is non-specific and exhibits a significant increase in concentration over a brief period of time (typically 6-12 h) [32]. As an outcome, many academics regularly utilize it to gauge how infected a patient is.

In the study of precise and rapid identification of sepsis with severe trauma, Li et al. [33] reported that CRP is an independent predictor of sepsis. In the opinion of Shi et al. [34], CRP has a mediocre level of efficacy in determining sepsis brought on by a new coronavirus infection. It was found that CRP was an independent risk factor for the emergence of pyonephrosis and was essential in the construct of the ML-assisted diagnostic model of calculous pyonephrosis designed by Liu et al. [18].

CRP is also the most significant factor in our research. In the testing set, the median (Q1, Q3) of CRP for the pyonephrosis group was 95.41 (56.53, 154.69) and the hydronephrosis group was 9.53 (4.98, 40.76). A noteworthy distinction was observed (*p* < 0.001). The AUC of CRP in the diagnosis of pyonephrosis was 0.82 (95%*CI*, 0.763-0.878) at the ideal cut-off value of 38.83. The corresponding accuracy, sensitivity, and specificity were 72.15%, 84.62% and 73.58%, respectively.

The connection between infection and hemoglobin is complicated. The immune system, inflammatory response, anemia and other factors are all involved in their interplay. During an infection, hemoglobin levels may drop, particularly in situations of severe or protracted infections. The body’s control of metabolism and inflammatory response are mostly to blame for this. Anemia can result from the body’s secretion of cytokines during an illness, including IL-1 and IL-6, which can prevent the bone marrow from producing RBC [35].

Second, when the body releases IL-6, it will cause a decrease in ferritin production and an increase in transferrin production. This may cause the body to use iron less effectively, which would have an impact on hemoglobin production. Furthermore, an elevation in the body’s metabolic rate may result in heightened dietary requirements. Anemia, which lowers hemoglobin levels, can result from the body not getting enough nutrition to satisfy its needs [36]. As result, while diagnosing pyonephrosis, hemoglobin level has a reference value. Along with treating the illness itself, patients’ nutritional state and anemia should be taken into consideration, and appropriate action should be done to maintain or improve the hemoglobin level of patients.

#### Urine characteristics

In terms of clinical diagnosis, pyonephrosis is highly dependent on urine infection. It’s important to note that in the training set, there were differences in urine WBC (*p* = 0.01) between the pyonephrosis and hydronephrosis groups, and in the testing set, there were differences in urine WBC (*p* < 0.001), urine culture (*p* = 0.17), urine nitrite (*p* = 0.34), and urinary bacterial count (*p* = 0.019). In addition to the aforementioned attributes, the model creation procedure incorporated the urine bacterial count, while the remaining features were eliminated during the screening phase. The reason why the liquid in the renal pelvis is difficult to reach the bladder might be because of the severe urinary tract blockage that is frequently observed in calculous pyonephrosis [37].

#### Else characteristics

The tissue density in CT is measured in HU values of CT. By determining how well the tissue absorbs X-rays, the HU values of CT indicate the density of the tissue. Its definition is derived on a comparison of human tissue’s X-ray absorption capability with that of air and water [38]. For instance, kidney stones typically show up as high-density regions with HU values of CT much greater than those of the surrounding tissues. This aids medical professionals in the diagnosis and location, size and form of kidney stones. The type of fluids may be identified by utilizing the HU value of effusion, which is an indication of a non-enhanced scan. The degree of higher HU value of effusion can indicate the severity of infection [39], which is the fundamental idea of HU value of effusion to discriminate between water and pus [40]. Infectious substances, cell debris and microorganisms in the liquid can cause an increase in HU value of effusion.

Although the predictive efficacy varies, the HU value of effusion is commonly utilized. The HU value of effusion of pyonephrosis was found to be substantially greater than that of hydronephrosis (13.51 ± 13.29 vs. 4.67 ± 5.37) when Yuruk et al [40] assessed the renal pelvis effusion in 105 individuals. The appropriate diagnosis of pyonephrosis had a sensitivity of 64.96% and a specificity of 87-93% when the HU value of effusion was less than 9.21, AUC of 0.780 ± 0.047 (95%*CI*, 0.689-0.855). There were 53 patients in Basmaci’s study [41]. Following data analysis, the groups with hydronephrosis and pyonephrosis had median HU value of effusion of -8.5 and 10, respectively, which accuracy of pyonephrosis was 100% and 96%, when the cut-off value was 0. Erdogan et al [42] verified that pyonephrosis and hydronephrosis could be distinguished from one another with a notable advantage using HU value of effusion. The diagnosis of pyonephrosis had a 68.4% sensitivity and a 92.6% specificity while the cutoff value was 8.46.

There was a substantial difference between the two groups in the present study, with the HU value of effusion in the hydronephrosis group being 2.44 ± 7.05 and the pyonephrosis group being 10.1 ± 7.78. The AUC, accuracy, sensitivity and specificity for the diagnosis of pyonephrosis were 70.9%, 84.60% and 69.81%, when the ideal cut-off value was 5.5.

Diabetes, being a systemic illness, can easily cause and exacerbate infection as well as lower immunity. Upper urinary tract stone development is associated with poor blood glucose management, according to research by Weinberg et al [43]. We also discovered that diabetes is a risk factor on its own. Thus, active blood glucose management has a favorable impact on preventing pyonephrosis and the formation of stones.

According to Wang et al [44], gender differences include the fact that urolithiasis is more frequent in males and that women are more likely than men to suffer from a urinary tract infection due to differences in anatomy. Since women’s urethras are shorter and straighter, they are more prone to urinary tract infections. Gender was also significantly different (*p* < 0.05) between the two groups in our study’s training and testing sets, despite it was not an independent risk factor for pyonephrosis.

#### Analysis of models results

Recent studies have demonstrated that ML is more effective than conventional statistical techniques in facilitating the generation of diagnoses for a range of illnesses [45]. ML has the potential to greatly expand computers’ capacity for “learning” without explicit programming and for handling large amounts of data through intricate interactions. Despite not being grounded in causal inference like traditional statistical approaches are, ML algorithms remain an essential tool for estimating causal effects in observational research. ML has the potential to improve outcomes by reducing bias, automatically managing missing features, minimizing raw data processing, controlling confounding, and balancing data [46].

Additionally, ML works effectively at assessing “big data” problems that are challenging for traditional statistical approaches to handle. Thus, automated systems for illness prediction, decision support, and detection of diseases that may arise in the population may be constructed with the assistance of ML techniques.

According to the findings, the GBDT model performed satisfactorily in predicting pyonephrosis, with LR coming in second. In this study, the LR model is deemed the most suitable due to its clinical interpretability and operability, which the accuracy, sensitivity and specificity of LR in predicting pyonephrosis were 0.873, 0.923 and 0.887. The LR model also demonstrated the highest stability, with an AUC of 0.957 on the testing set compared to 0.938 on the training set. In contrast, Wang et al [23] found preoperative diagnosis of calculous pyonephrosis vary in that the AUC of the remaining four models on the testing set is lower than that on the training set. Compared to the training set, all of their models exhibit worse performance on the testing set.

In general, the performance of the model on the training set should be better than or equal to that on the test set when the generalization ability of the model is excluded. This is because the model performs parameter adjustment and fitting on the training set, which may be more suitable for training data. Based on the results of LR model prediction, we analyze it from the following three aspects. First, disparities in data distribution. Data distributions may differ across the testing and training sets. The dataset features that were used for the construction and validation of the model are essentially the same, and the balance is good, according to the difference test, which revealed that there was no significant difference in any of the characteristics of the pyonephrosis and hydronephrosis groups in the training set and the testing set (*p* > 0.05). The second is data leaks. It is possible for data leakage to occur during feature engineering or data preprocessing, which would improve the model’s performance on the training set. We revealed there was no the testing set data during the LR model building process. Instead, we fit the model only using the training set. Thirdly, the assessment method. For example, the division of a single training set and a testing set may be affected by randomness. Alternatively, we had attempted to split the training and testing sets into 8:2 and 6:4 in place of the random number of seeds. In spite of this, the testing set’s AUC for the model remains greater than the training set. Based on this, we believe that the LR model has significant predictive power and great stability.

Due to its outstanding predictive accuracy, the model is expected to be used to assist physicians identify patients suffering from hydronephrosis and pyonephrosis and decide whether to treat the infection or do an ultrasound-guided percutaneous renal puncture and drainage right away. This has immense importance in enhancing the efficacy and productivity of patient care.

Our findings have practical implications for the early diagnosis of pyonephrosis. Future research should concentrate on multi-center prospective data to increase research samples, add more clinical features or image data such as radiomics. It should also continue to optimize the model’s parameters or use deep learning techniques to develop a decision support system that is integrated into the electronic medical record system, thereby improving the practical application of this technology. This should be done since medical technology is always evolving.

Our model still has certain restrictions even with its good performance. Initially, there is a low rate of conversion in clinical use. Both the prediction model’s accuracy and its simplicity must be taken into consideration when using it in a clinical setting (TNM, for example). Second, the model is built and verified using a single-center retrospective data set. The verification process is not done using prospective data, nor is there multi-center data. The model’s application value is restricted and its stability may be weak. Third, the study’s sample size is tiny, and more data is needed to further train and validate the model’s accuracy.

## Conclusion

By using ML to create a diagnostic prediction model, calculous pyonephrosis was predicted with a considerable increase in accuracy when compared to traditional analytic approaches. The most significant characteristic is CRP, which was chosen Lasso regression from a set of 43 clinical parameters. The other characteristics were renal colic, diabetes, hemoglobin, IL-6, urine bacterial count, globulin and G/A. The top five important characteristics in the LR, XGBoost, RF and GBDT models include CRP, renal colic and HU value of effusion. The GBDT model has the greatest AUC out of the five models, with the LR model coming in second. Among the five models, LR and GBDT models have the same prediction accuracy, but the sensitivity and specificity are the highest for the LR model. In our study, the LR model is deemed the best model combine to its superior clinical operability or interpretability. In clinical practice, urologists may find the LR model nomogram to be a useful supplementary tool in properly diagnosing pyonephrosis.

## Data Availability

All relevant data are within the paper.
